# Synergistic Enhancement of Non-viral SIRT1 Gene Delivery to Human Skin Equivalents Using Microneedling and Liposomal Vectors

**DOI:** 10.7759/cureus.101876

**Published:** 2026-01-19

**Authors:** Wataru Matsunaga, Takahiro Ishikawa, Miki Nakashima, Akinobu Gotoh

**Affiliations:** 1 Joint-Use Research Facilities, Hyogo Medical University, Nishinomiya, JPN; 2 Affiliated Research Institute, Foundation for Kobe International Medical Alliance, Kobe, JPN; 3 Research Department, GCT (Global Connector Technology) Co. Ltd., Kasuyamachi, JPN; 4 Department of Education for Medical Research Base, Hyogo Medical University, Nishinomiya, JPN

**Keywords:** gene delivery, liposomes, microneedling, sirt1, skin aging

## Abstract

Introduction: The "longevity gene" sirtuin 1 (SIRT1) is a critical regulator of oxidative stress and cellular senescence, making it a high-value target for anti-aging dermatological interventions. However, the stratum corneum poses a formidable barrier to the transdermal delivery of therapeutic genes. This study systematically evaluated the efficacy of non-viral SIRT1 plasmid delivery to a full-thickness 3D human skin equivalent (T-Skin™) using distinct vector formulations and physical enhancement methods.

Materials and methods: We compared the delivery efficiency of naked plasmid DNA and a cationic liposomal formulation using two delivery methods: (1) simple topical application and (2) automated microneedling (Dermapen, 0.2 mm diameter). SIRT1 expression was quantified 24 hours post-delivery using quantitative reverse transcription polymerase chain reaction (RT-qPCR) and validated by immunohistochemistry (IHC).

Results: The results demonstrated a pronounced synergistic effect: the combination of a liposomal plasmid with Dermapen (Lipo-Dermapen) yielded the highest efficiency, with a ~357-fold increase in SIRT1 mRNA expression compared with the control. Notably, the second-highest efficiency was achieved by the simple, non-invasive, and topical application of naked plasmids (Plasmid-Direct), which achieved a remarkable ~70-fold increase in expression. This non-invasive approach unexpectedly outperformed other delivery groups, including liposomal topical application (Lipo-Direct, ~27-fold) and naked plasmid with Dermapen (Plasmid-Dermapen, ~10-fold).

Conclusions: The Lipo-Dermapen platform provides robust proof of concept for the development of high-efficacy, minimally invasive clinical gene therapies. Furthermore, the unexpected efficacy of non-invasive naked plasmid delivery suggests a novel potential for accessible, self-administered dermatological interventions.

## Introduction

In the field of cosmetic dermatology, there is a growing demand for effective and minimally invasive rejuvenation treatments to address the complex processes of skin aging [1,2]. Skin aging is a multifactorial biological phenomenon driven by intricate mechanisms, including the progressive decline in dermal fibroblast function, which leads to a significant reduction in extracellular matrix components, particularly collagen levels. Concurrently, the cumulative effects of oxidative stress and weakened epidermal barrier function contribute to the visible and structural decline in skin homeostasis [3].

Addressing these underlying biological drivers is a primary objective in developing next-generation dermatological interventions. Consequently, there is an active and expanding search for novel molecular targets capable of counteracting these aging processes and restoring skin homeostasis.

Sirtuin 1 (SIRT1), a nicotinamide adenine dinucleotide (NAD+)-dependent protein deacetylase, has emerged as a promising therapeutic target for anti-aging [4,5]. SIRT1 also plays a pivotal role in skin aging by modulating fundamental cellular processes, including the mitigation of oxidative stress, promotion of DNA repair, and protection of dermal fibroblasts from cellular senescence [6]. Therefore, SIRT1 is a key mediator in counteracting both chronological and photoaging processes, and developing a robust methodology for localized upregulation of SIRT1 expression represents a significant therapeutic and aesthetic objective for skin rejuvenation.

Physical enhancement methods and vector-based systems are widely considered strategies for enhancing transdermal delivery. Physical methods, such as mechanical disruption of the stratum corneum with microneedling [7,8] and perturbation of cell membranes using electrical fields via electroporation [9], are established techniques for enhancing the permeability of macromolecules in the skin. Concurrently, vector-based strategies, particularly liposomal encapsulation [10], have been employed to protect nucleic acid payloads from nuclease degradation and facilitate cellular uptake. Although these platforms have been investigated independently, the optimal synergistic combination of physical and vector-based approaches for non-viral plasmid DNA delivery to the skin remains undefined.

In this study, we systematically investigated the synergistic efficacy of physical vector platforms for SIRT1 plasmid delivery using T-Skin^TM^, a full-thickness 3D human skin equivalent model [11]. We quantitatively compared the delivery efficiency, assessed by SIRT1 mRNA expression via quantitative RT-PCR (RT-qPCR), and validated the protein expression and localization using immunohistochemistry (IHC). This study aimed to identify and mechanistically evaluate both non-liposomal and liposomal-formulated SIRT1 plasmids delivered by simple topical application and a 0.2 mm automated microneedling device to validate an optimized platform for efficient non-viral gene delivery for future therapeutic and cosmetic applications.

## Materials and methods

Tissue model and culture

A full-thickness 3D human skin equivalent, T-Skin^TM^ (EpiSkin, Lyon, France), was used for all experiments. This model consists of a dermal equivalent containing human fibroblasts overlaid with a stratified, differentiated epidermis derived from normal human keratinocytes [11]. The T-Skin was stored at room temperature, and acclimatization was initiated within one week. The T-Skin was aseptically transferred to six-well plates containing 2 mL of pre-warmed medium and incubated overnight at 37°C in 5% CO_2_. The medium was changed daily, and all experiments were initiated within 48 hours of acclimatization.

Plasmid and liposome preparation

**Figure 1:**
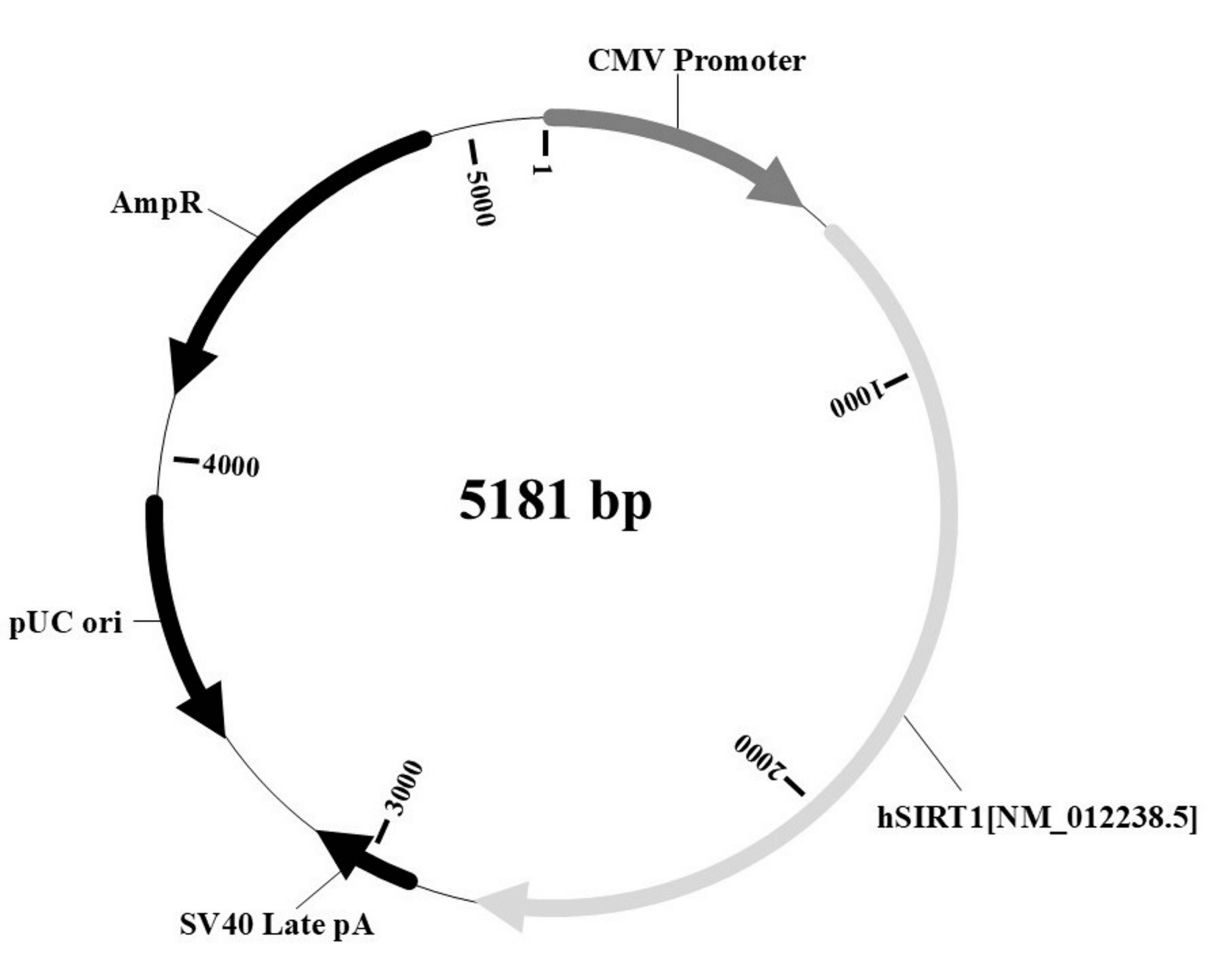
Schematic of the SIRT1 plasmid. hSIRT1: Human SIRT1 gene (NM_012238.5); CMV promoter: Human cytomegalovirus immediate early enhancer/promoter; SV40 Late pA: Simian virus 40 late polyadenylation signal; pUC ori: pUC origin of replication; AmpR: Ampicillin resistance gene (Drawn by the author based on the information from VectorBuilder.)

The SIRT1-encoding plasmid used in this experiment was obtained from VectorBuilder (Chicago, IL, USA) (Figure 1). The plasmid backbone included a human SIRT1 gene (NM_012238.5) driven by a CMV promoter, with an SV40 Late polyadenylation signal and an ampicillin resistance gene (AmpR) for bacterial selection.

For the preparation of empty cationic liposomes, 1,2-dimyristoyl-sn-glycero-3-phosphocholine (NOF Corporation, Tokyo, Japan) and N-(α-trimethylammonioacetyl)-didodecyl-D-glutamate chloride (Sogo Pharmaceutical Co., Ltd, Tokyo, Japan) were dissolved in water for injection (Nissin Pharmaceutical, Tendo, Japan) and sonicated (UP400St, Hielscher Ultrasonics, Teltow, Germany) at 45°C for 40 minutes. The mixture was then filtered through a 0.22 μm membrane.

The concentration of the SIRT1 plasmid was determined by measuring nucleic acid concentration using a NanoDrop One spectrophotometer (Thermo Fisher Scientific, Waltham, MA). Based on this measurement, the plasmid solution was adjusted to a final concentration of 60 ng/µL for all experiments.

Two formulations of SIRT1-encoding plasmids were then prepared. For the liposomal plasmid (Lipo-SIRT1), the adjusted plasmid solution was mixed with an equal volume of cationic liposome suspension (0.5 mg/mL) by rapid pipetting. For naked SIRT1, the plasmid solution was diluted 1:1 with Tris-EDTA buffer to match the dilution applied during liposomal complex formation, thereby equalizing the final plasmid concentration between formulations (final concentration: 30 ng/µL). For each sample, a volume of 50 µL of the prepared formulation (corresponding to 1.5 µg of plasmid DNA) was applied, ensuring an identical amount of SIRT1 plasmid across all experimental groups.

Experimental groups and delivery protocols

T-Skins (N = 2 per group) were treated with 50 µL of Lipo-SIRT1 or Naked SIRT1 using one of the following delivery methods. In the "Direct" (non-invasive) group, Lipo-SIRT1 and Naked SIRT1 (50 µL each) were applied topically and spread using a sterile glass spreader. In the "Micro-invasive" (microneedling) group, the surface of the T-Skin was pre-treated using an automated microneedling device (Dermapen Pro, DermapenWorld, Sydney, Australia). The needle depth was set to 0.2 mm, and the speed was set to 7000 rpm. Immediately thereafter, 50 µL of Lipo-SIRT1 or Naked SIRT1 was applied topically and spread using a sterile glass spreader. Untreated T-Skin samples served as a negative control.

Following plasmid delivery, all samples were incubated for 24 hours at 37°C in 5% CO_2_.

The 24-hour point was selected to capture early transgene expression after cutaneous nucleic acid delivery, which has been reported to reach maximal or near-maximal levels around 24 hours in skin following intradermal gene delivery approaches [12], and has also been used to evaluate microneedle-facilitated plasmid expression in viable human skin [13].

The T-Skin samples were then bisected; one half was fixed in 10% formalin for histological processing, and the other half was flash frozen in liquid nitrogen for RT-qPCR analysis.

RNA extraction and quantitative real-time PCR

For quantitative RT-PCR (RT-qPCR), the total RNA was extracted from homogenized T-Skin samples using a nucleospin RNA extraction kit (Macherey-Nagel, Düren, Germany) according to the manufacturer's protocol.

Quantitative real-time PCR (qPCR) was performed by a one-step method with RNA-Direct® SYBR™ Green Realtime PCR Master Mix (QRT-201, TOYOBO, Osaka, Japan). The thermal cycling conditions were as follows: initial denaturation at 90°C for 30 seconds, reverse transcription at 61°C for 20 minutes, and second denaturation at 95°C for one minute. This was followed by 45 cycles of denaturation at 95°C for 15 seconds, annealing at 54°C for 15 seconds, and extension at 74°C for 45 seconds. A Rotor-Gene Q thermal cycler (Qiagen, Hilden, Germany) was used for PCR.

The primers used in this study were purchased from Integrated DNA Technologies (Coralville, IA, USA). The primer sequences were as follows: *SIRT1* forward: 5'-TCG CAA CTA TAC CCA GAA CAT AGA CA-3' *SIRT1* reverse: 5'-CTG TTG CAA AGG AAC CAT GAC A-3' Glyceraldehyde-3-phosphate dehydrogenase (*GAPDH*) forward: 5'-GAA AGC CTG CCG GTG ACT AAC C -3' *GAPDH* reverse: 5'-CCC GGA GGA GAA ATC GGG C-3’. The mRNA expression of SIRT1 was quantified by the comparative Ct method (ΔΔCt method) relative to the expression of *GAPDH*.

Data analysis

*SIRT1* mRNA expression was quantified by RT-qPCR (N = 2 replicates per sample) and normalized to the housekeeping gene *GAPDH*. Relative gene expression was determined by calculating the fold change (2^-ΔΔCt^) relative to that of the negative control group. Data are presented as mean fold change values. Given the proof-of-concept nature of this study using a standardized industrial tissue model and the distinct magnitude of the observed differences, descriptive evaluation was prioritized over hypothesis testing.

Immunohistochemistry and image analysis

For the immunohistochemistry (IHC) analysis, formalin-fixed, paraffin-embedded, 3 µm thick T-Skin sections were processed using the Ventana BenchMark XT auto-immunostainer (Roche, Basel, Switzerland) and Ventana ultraView DAB universal kit (Roche). The sections were pre-incubated with 5% skim milk containing Ez buffer (ATTO, Tokyo, Japan) for 10 minutes at room temperature. After deparaffinization, antigen retrieval was performed using a Cc1 antigen retrieval buffer (pH 8.5, Roche). The sections were incubated with an anti-human SIRT1 antibody (x500, SART-1 Antibody sc-376460, Santa Cruz Biotechnology, Dallas, TX, USA) at 37°C for 34 minutes, followed by visualization using a kit-provided secondary antibody (multimer HRP-conjugated anti-mouse IgG) with HRP and 3,3'-diaminobenzidine (DAB). Finally, the nuclei were counterstained with hematoxylin.

Image analysis of SIRT1 expression was performed using the ImageJ/Fiji software (National Institutes of Health, Bethesda, MD, USA). To specifically quantify the intensity of DAB staining, color deconvolution (vector: H DAB) was applied, and the DAB-specific channel (Color_2) was selected for analysis [14]. The pixel intensity values were converted to optical density (OD) according to the following formula:

$OD = log_{10}(\frac{255}{pixel value})$

Regions of interest (ROIs) were manually drawn around individual positive cells, and the mean OD value was calculated for each ROI.

## Results

Gene expression analysis

All four delivery methods (Direct + naked SIRT1, Dermapen + naked SIRT1, Direct + Lipo-SIRT1, and Dermapen + Lipo-SIRT1) resulted in successful SIRT1 gene expression, with mRNA levels significantly higher than the baseline endogenous expression observed in the negative control group (average Ct of SIRT1 mRNA was 25.62). The housekeeping gene GAPDH was stably expressed across all groups (average Ct range = 25.17 to 29.89), validating its use for normalization.

The relative quantification of expression revealed a wide and distinct range of efficiencies, which are summarized in Table 1. The combination of Dermapen+Lipo-SIRT1 was the most effective, yielding a ~357-fold increase in SIRT1 mRNA expression compared to the control.

**Table 1:** Relative quantification of SIRT1 mRNA expression (24-hour post-delivery) ΔΔCt was calculated relative to the Negative Control. The fold change was calculated as 2^−ΔΔCt^. Ct: threshold cycle; GAPDH: glyceraldehyde-3-phosphate dehydrogenase; SIRT1: sirtuin 1; Avg: average; N/A: not applicable

Experimental group	Formulation	Delivery method	*SIRT1* (Avg Ct)	*GAPDH* (Avg Ct)	ΔCt (*GAPDH-SIRT1*)	ΔΔCt (vs. control)	Fold change
Negative control	N/A	N/A	25.62	29.89	4.27	0	1 (100%)
Direct	Naked SIRT1	Non-invasive (topical)	14.78	25.18	10.4	6.13	~70.0 (7,000%)
Dermapen	Naked SIRT1	Micro-invasive	18.11	25.77	7.66	3.39	~10.0 (1,000%)
Direct	Lipo-SIRT1	Non-invasive (topical)	16.17	25.17	9	4.73	~27.0 (2,700%)
Dermapen	Lipo-SIRT1	Micro-invasive	14.92	27.67	12.75	8.48	~357.0 (35,700%)

The second most effective method was Direct + naked SIRT1, which resulted in a ~70-fold increase. This non-invasive method unexpectedly outperformed all other enhanced delivery groups, including Direct + Lipo-SIRT1 (~27-fold) and Dermapen + naked SIRT1 (~10-fold).

Histological analysis

**Figure 2:**
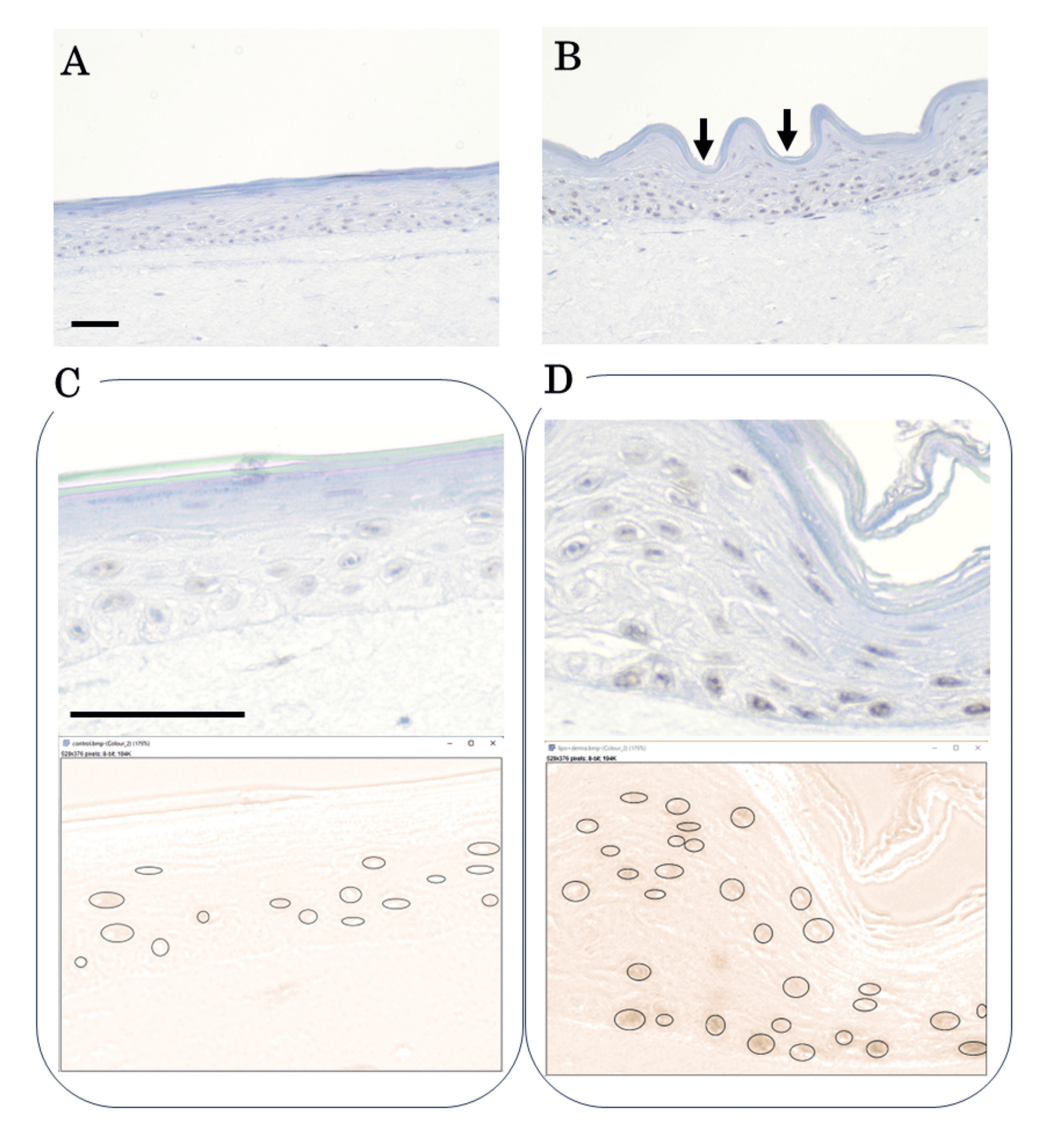
Expression of SIRT1. (A) Immunohistochemical section of the control group, showing faint baseline SIRT1 staining. (B) Lipo-SIRT1 + Dermapen group. The epidermis exhibits a characteristic "undulating" morphology induced by microneedling. Intense SIRT1 expression (brown) was localized in the "valleys" (needle insertion sites). (C, D) Representative images of the semi-quantitative analysis for the control (C) and Lipo-SIRT1 + Dermapen (D) groups. The circles in the lower panel indicate the regions of interest (ROIs) manually selected for calculating the OD values. Scale bar = 200 μm.

IHC analysis was performed to confirm that the observed increase in mRNA levels was successfully translated into SIRT1 protein expression (Figure 2). Hematoxylin staining of the epidermis in the Dermapen + Lipo-SIRT1 group revealed a distinct “undulating” morphology (Figure 2B). This confirmed the physical action of the 0.2 mm microneedles, which created microchannels in the tissue.

The IHC results showed that the negative control tissue exhibited only faint, diffuse baseline staining for SIRT1 (Figure 2A), with an average OD value of DAB staining of 0.0460 ± 0.011 (Figure 2C). In contrast, the Dermapen + Lipo-SIRT1-treated tissue showed intense dark brown positive staining for SIRT1 (Figure 2B). Moreover, these immune-positive signals were localized in the "valleys" of the wavy tissue, corresponding directly to the microchannels created by Dermapen. Immune staining was weak in the "wave peaks" or intact tissue where the needles did not penetrate. Expression was most prominent at the dermal-epidermal junction. The average OD value of DAB staining for the treated group was 0.0777 ± 0.0187 (Figure 2D).

## Discussion

The SIRT1 gene, a key regulator of cell survival and longevity, is a prime target for advanced anti-aging interventions. In this study, we identified two distinct and viable platforms for delivering the SIRT1 gene to the skin, with direct implications for future cosmetic applications.

The primary finding of this study was the remarkable ~357-fold synergistic effect of combining a liposomal vector with microneedling (Dermapen + Lipo-SIRT1). This result mechanistically addresses two fundamental barriers to transdermal gene delivery. First, the Dermapen, set to 0.2 mm, physically creates micro-conduits through the stratum corneum, bypassing the primary barrier of the skin. The IHC results, showing protein expression localized only in the needle tracts, suggest that providing a physical breach is a critical first step. Second, liposomal formulations protect plasmid DNA from nuclease degradation and facilitate its cellular uptake. This was strongly supported by the results of the Dermapen + Naked SIRT1 group (~10-fold). We hypothesized that microtrauma from Dermapen releases extracellular nucleases that rapidly destroy unprotected naked plasmids.

The liposome provided the necessary protection to exploit the physical channel, leading to a remarkable increase in the signals. This platform represents an ideal model for a high-efficacy, minimally invasive clinical cosmetic treatment in which a professional administers gene therapy using a microneedling device. However, given that microneedling devices are publicly accessible and raise potential safety concerns regarding self-administration, the development of more specialized, clinic-only equipment may be desirable.

In parallel, high transfection efficiency can be achieved even with a non-invasive gene transfer method. The most unexpected but notable finding was the success of the "non-invasive" Direct + Naked SIRT1 group, which yielded a ~70-fold increase in SIRT1 mRNA expression. This simple topical application of naked DNA was seven times more effective than the application of naked DNA with a Dermapen (~10-fold) and more effective than the topical application of liposomal SIRT1 (~27-fold).

The delivery of naked plasmid DNA via topical application to intact skin has been previously documented [15], with expression localized to the epidermis and hair follicles [16]. However, a critical distinction in our study is the use of the T-Skin model, which is a reconstructed tissue devoid of hair follicles. If the structural integrity of T-skin is less robust than that of living skin, the friction from the glass spreader likely created superficial micro-abrasions, compromising the barrier function sufficiently to allow the passive diffusion of the plasmid. Furthermore, the naked plasmid has a significantly smaller hydrodynamic radius compared to the liposomal complex. The liposomal vector used in this study was likely optimized for in vivo uptake (e.g., endocytosis) but poorly optimized for passive topical penetration. Its larger size or charge may have inhibited its diffusion through the stratum corneum, making it less effective than smaller naked plasmids for simple topical application. However, this hypothesis cannot explain why the Dermapen naked SIRT1 showed lower expression results, and further investigation is required.

Regardless, the finding that noninvasive topical application (plasmid-direct) achieved significant gene delivery in a standardized epithelial barrier model is particularly noteworthy. This suggests that with further vector optimization, a similar noninvasive topical approach could be explored for other epithelial barriers. For example, topical eye drops that deliver the SIRT1 gene without ocular damage could represent a novel therapeutic avenue for conditions such as glaucoma, where SIRT1 is known to be a potent neuroprotective target for retinal ganglion cells [17]. This approach would bypass the significant risks associated with invasive intraocular injections.

This study provides a proof-of-concept for two distinct therapeutic approaches. The Lipo-Dermapen platform is a promising candidate for clinical development, potentially utilizing commercially available equipment under professional supervision. Conversely, the "direct-plasmid" finding may open a novel path: the development of an "at-home" topical gene therapy for cosmetic applications. Future research should focus on optimizing a vector designed for passive topical penetration, potentially combining the high expression of a protected plasmid with the ease of non-invasive application.　

A limitation of this study is the sample size of N = 2 biological replicates. However, we considered that the use of the T-Skin model, an industrially standardized human skin equivalent, ensures high reproducibility compared to heterogeneous ex vivo tissues. Furthermore, the magnitude of the observed effect (357-fold increase) significantly exceeds standard biological variance, and we therefore believe the results of this study are valid and informative.

## Conclusions

This study successfully validated a highly effective platform for non-viral SIRT1 gene delivery to human skin equivalents. The synergistic combination of liposomal encapsulation and microneedling (Dermapen) resulted in a ~357-fold increase in gene expression, confirming its potential as a high-efficacy clinical platform for cosmetic anti-aging treatments. In addition, the unexpected discovery that a simple, non-invasive topical application of naked plasmid DNA yielded a robust ~70-fold increase in expression opens a novel and exciting pathway for developing at-home, topical gene therapies. These findings provide a strong foundation for the future development of gene-based cosmetic interventions.
